# *Trichoderma* Species Differ in Their Volatile Profiles and in Antagonism Toward Ectomycorrhiza *Laccaria bicolor*

**DOI:** 10.3389/fmicb.2019.00891

**Published:** 2019-04-26

**Authors:** Yuan Guo, Andrea Ghirardo, Baris Weber, Jörg-Peter Schnitzler, J. Philipp Benz, Maaria Rosenkranz

**Affiliations:** ^1^Research Unit Environmental Simulation (EUS), Institute of Biochemical Plant Pathology, German Research Center for Environmental Health, Helmholtz Zentrum München, Neuherberg, Germany; ^2^Holzforschung München, TUM School of Life Sciences Weihenstephan, Technical University of Munich, Freising, Germany

**Keywords:** *Trichoderma*, volatile organic compounds, fungi–fungi interaction, antagonism, beneficial fungi, ectomycorrhiza, *Laccaria bicolor*, inhibition

## Abstract

Fungi of the genus *Trichoderma* are economically important due to their plant growth- and performance-promoting effects, such as improved nutrient supply, mycoparasitism of plant-pathogens and priming of plant defense. Due to their mycotrophic lifestyle, however, they might also be antagonistic to other plant-beneficial fungi, such as mycorrhiza-forming species. *Trichoderma* spp. release a high diversity of volatile organic compounds (VOCs), which likely play a decisive role in the inter-species communication. It has been shown that *Trichoderma* VOCs can inhibit growth of some plant pathogens, but their inhibition potentials during early interactions with mutualistic fungi remain unknown. *Laccaria bicolor* is a common ectomycorrhizal fungus which in symbiotic relationship is well known to facilitate plant performance. Here, we investigated the VOC profiles of three strains of *Trichoderma* species, *Trichoderma harzianum, Trichoderma Hamatum*, and *Trichoderma velutinum*, as well as *L. bicolor* by stir bar sorptive extraction and gas chromatography – mass spectrometry (SBSE-GC-MS). We further examined the fungal performance and the VOC emission profiles during confrontation of the *Trichoderma* species with *L. bicolor* in different co-cultivation scenarios. The VOC profiles of the three *Trichoderma* species were highly species-dependent. *T. harzianum* was the strongest VOC emitter with the most diverse compound pattern, followed by *T. hamatum* and *T. velutinum*. Co-cultivation of *Trichoderma* spp. and *L. bicolor* altered the VOC emission patterns dramatically in some scenarios. The co-cultivations also revealed contact degree-dependent inhibition of one of the fungal partners. *Trichoderma* growth was at least partially inhibited when sharing the same headspace with *L. bicolor*. In direct contact between both mycelia, however, *L. bicolor* growth was impaired, indicating that *Trichoderma* and *L. bicolor* apply different effectors when defending their territory. Multivariate analysis demonstrated that all examined individual fungal species in axenic cultures, as well as their co-cultivations were characterized by a distinct VOC emission pattern. The results underline the importance of VOCs in fungal interactions and reveal unexpected adjustability of the VOC emissions according to the specific biotic environments.

## Introduction

Biogenic volatile organic compounds (BVOCs) are a large group of chemically diverse small molecules emitted by plants, microbes, and fungi. Plant VOCs have well described functions in communication, interaction, and defense especially aboveground (Baldwin et al., 2006; Šimpraga et al., 2016). Belowground, the role of VOCs during plant-microbe interactions has only recently been recognized (Penuelas et al., 2014). Due to their volatility and their diffusiveness through air and liquid spaces, VOCs have ideal biophysical properties to function as signaling molecules aboveground but also belowground via pores in the soil matrix (Schulz-Bohm et al., 2017). So far, VOC emission profiles from around 600 microbial and fungal species have been obtained (Schulz-Bohm et al., 2017). Fungi emit a large spectrum of VOCs including acids, alcohols, aldehydes, aromatics, esters, heterocycles, ketones, thiols (Hung et al., 2015), and highly reactive terpenes (Weikl et al., 2016). Terpenes, and particularly SQTs, that fungi are a significant source of, play important roles also in atmospheric chemistry (Riipinen et al., 2012; Bourtsoukidis et al., 2018).

Recently, several research groups made intensive efforts to study the ecological and biological roles of fungal VOCs (Penuelas et al., 2014; Schenkel et al., 2015; Werner et al., 2016). Considering that over 5 million fungal species are predicted to live on earth (Blackwell, 2011), there is tremendous potential to find novel fungal bioactive compounds that are important in belowground interactions (Piechulla and Degenhardt, 2014). Especially the function of volatiles released from plant-beneficial fungi, such as symbiotic mycorrhizal and plant growth-promoting fungi (PGPF) has been of major interest (Morath et al., 2012; Ditengou et al., 2015; Schenkel et al., 2015). Fungal VOCs can aid plants by priming and activation of defense responses (Kishimoto et al., 2006, 2007; van Hulten et al., 2006), providing growth promotion of nearby plants (Morath et al., 2012), as well as by direct growth inhibition of phytopathogens (Strobel et al., 2001).

*Trichoderma* spp. are well-known PGPF having an ability to compete against pathogenic microbes and to promote plant fitness (Bitas et al., 2013). The genus *Trichoderma* comprises 254 identified species and 2 varieties (Bissett et al., 2015) which are ubiquitously present in forest and agricultural soils, where they are highly interactive with plant roots and rhizospheric microorganisms (Harman et al., 2004). Studies showed that *Trichoderma* spp. can act as elicitors promoting plant health by priming against pathogens (Bigirimana et al., 1997; Harman et al., 2004). In addition to the well-recognized induction of systemic and local plant immunity (Verma et al., 2007), *Trichoderma* species have been demonstrated to act as biological control agents for many soil-borne pathogens including several plant-pathogenic fungi, such as *Armillaria, Chondrostereum, Phytophthora, Rhizoctonia, Sclerotinia, Verticillium*, and others (Monte, 2001; Contreras-Cornejo et al., 2016).

The genomes of several *Trichoderma* species have been identified as being rich in genes encoding enzymes responsible for secondary metabolite production, which may contribute to a potential competitive advantage in their biocontrol activities, and of which those encoding for VOCs are an important subset (Kubicek et al., 2011; Mukherjee et al., 2012). Some studies have exploited the VOCs of *Trichoderma* spp., showing that the emission profiles depend on species/strains, substrate composition, and cultivation environment (Stoppacher et al., 2010; Crutcher et al., 2013). So far, approximately 480 different VOCs have been detected from *Trichoderma* species altogether. The detected *Trichoderma* VOCs comprise simple hydrocarbons, heterocycles, aldehydes, ketones, alcohols, phenols, thioalcohols, thioesters, and their derivatives (reviewed by Siddiquee, 2014). Some of these VOCs were shown to be detrimental to plant pathogens, indicating that VOCs may play a role in the biocontrol activity of *Trichoderma* spp. (Morath et al., 2012; Contreras-Cornejo et al., 2014; Li et al., 2018), and several studies have indicated an inhibitory effect of *Trichoderma* VOCs on wood decay fungi (Srinivasan et al., 1993; Bruce et al., 1996; Wheatley et al., 1997). In addition, some *Trichoderma* VOCs were reported to induce plant resistance, (Kottb et al., 2015) and to directly promote plant growth (Hung et al., 2013; Lee et al., 2016, 2019; Nieto-Jacobo et al., 2017).

In the present study, we explored and compared VOC emissions of three commercially relevant *Trichoderma* species: *T. harzianum, T. hamatum*, and *T. velutinum*. So far, no information exists on VOCs of *T. hamatum* or *T. velutinum*, whereas the previous studies on *T. harzianum* VOCs showed high discrepancies (Wheatley et al., 1997; Hung et al., 2013; Siddiquee, 2014; see also discussion). All three species are known mycoparasites on phytopathogenic fungi (Hung et al., 2013; Sharma et al., 2017), but so far, no studies have analyzed their performance in presence of other mutualistic fungi. *Laccaria bicolor* is an ectomycorrhizal fungus found throughout the temperate zones of the world and forms a symbiosis with several conifer roots (Courty et al., 2009) as well as, e.g., with *Populus* spp. (Plett et al., 2015). Also, *Trichoderma* spp. can be associated with *Salix* spp. and *Populus* spp. (Wuczkowski et al., 2003). Given their common occurrence and the overall benefits when these fungi are used for growth promotion purposes, their interactions are of interest and warrant a detailed investigation. While a few confrontation studies have been performed in the past (Summerbell, 1987; Rousseau et al., 1996; Werner et al., 2002), the results were mixed and the involvement of VOCs in the interactions between *Trichoderma* and mycorrhizal fungi is so far completely unknown. Within the present study, we thus examined different confrontation scenarios of the three *Trichoderma* species with *L. bicolor* and the involvement of VOCs in these interactions. Our results revealed distinct and species-dependent VOC emission profiles, which were found to be dynamically adjusted when *Trichoderma* was confronted with *L. bicolor*. Moreover, the antagonistic activities of the *Trichoderma* species were likewise unique, indicating a species-specific response to the mycorrhizal co-culture.

## Materials and Methods

### Fungal Strains and Cultivation

*Trichoderma harzianum* WM24a1, *T. hamatum* QL15d1, *T. velutinum* GL1561, and *L. bicolor* S238N strains were cultivated in a growth chamber with 23°C and permanent darkness on modified Melin-Norkrans synthetic medium (previously described by Müller et al., 2013). For VOC measurements and confrontation studies, fungal pieces of mycelium were punched out with a cork borer (1 cm diameter) and inoculated in glass Petri dishes (10 cm diameter) containing 40 ml modified Melin-Norkrans synthetic medium.

### Experimental Setup and Growth Analysis of the Fungi

**FIGURE 1:**
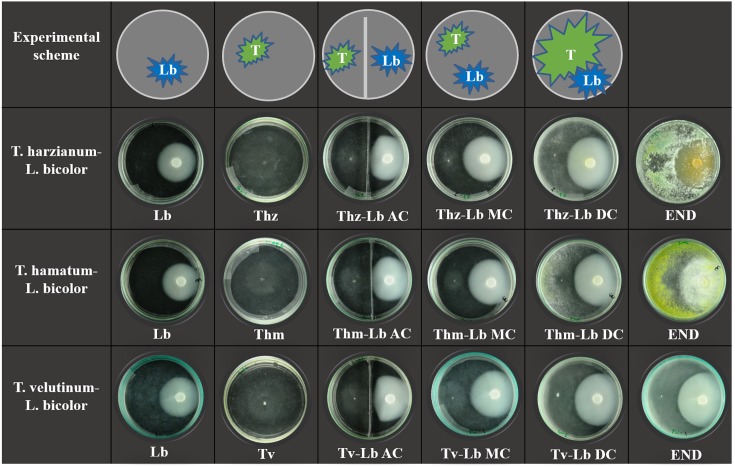
Scheme of experimental setup and culture morphology. The top panel shows the experimental scheme where T indicates *Trichoderma* species, Lb indicates *Laccaria bicolor*. Three lower panels display the actual morphology of the individual fungus and co-cultures, where Thz, *Trichoderma harzianum*; Thm, *Trichoderma hamatum*; Tv, *Trichoderma velutinum*; Lb, *Laccaria bicolor*. The degree of contact in co-cultivation conditions in bi-partial Petri dishes is indicated as NC, no contact; AC, airborne contact and in direct confrontation set ups as MC, media contact; DC, direct contact. END indicates the final status of *Trichoderma*–*L. bicolor* co-cultures in direct physical contact (9 days after inoculation with *Trichoderma* species).

Initially, *L. bicolor* was inoculated on normal glass Petri dishes (Non-Split, 10 cm diameter) and bi-compartment Petri dishes (10 cm diameter, separated by a metal strip). After 14 days of cultivation (fungal mycelium area at that time point was approximately 15 cm^-2^), VOCs were collected from *L. bicolor* alone. Subsequently, the fast-growing *T. harzianum, T. hamatum*, or *T. velutinum* mycelia were inoculated onto the same Petri dishes. On normal Petri dishes, the two fungi had contact through solid media (MC) and headspace, whereas in bi-compartment dishes only AC was possible. By the end of the experiment, the MC contact turned to direct physical contact (DC) between the two fungal species. Eight days after *Trichoderma* inoculation, final pictures were taken with co-cultivations in normal Petri dishes (end). The visualization of the set-up is shown in Figure 1.

Two days post *Trichoderma* inoculation, VOC collection was performed on all the co-cultivations as well as the individual fungi. When *Trichoderma* spp. and *L. bicolor* got into physical contact (3 days post inoculation with *Trichoderma*), VOCs were collected to examine VOC profiles under DC of the two species. Six replicates of each treatment and control were performed. Control Petri dishes only contained media. Prior to each VOC collection, pictures of fungal mycelium were taken with a Nikon D300 camera (60 mm Nikkor AF-S Micro-Nikkor Lens, Nikon, Tokyo, Japan). The growth inhibition was calculated using the following formula (Raut et al., 2014):

$$Growth\;Inhibition\;(\%)=\frac{D1-D2}{D1}\times 100;$$

Where D1 denotes the fungal area grown alone and D2 denotes the fungal area in co-cultivations.

### Analysis of VOCs

Volatile organic compounds were collected from the headspace of fungal cultures for 16 h at 23°C in the dark by stir bar sorptive extraction (SBSE) technique (Twisters, Gerstel GmbH & Co.KG, Mülheim an der Ruhr, Germany) as previously described (Müller et al., 2013). The twisters were fixed inside the top of the Petri dishes with a magnet placed outside (Weikl et al., 2016). The samples were analyzed by thermo desorption-gas chromatography-mass spectrometry (TD-GC-MS), and VOC analyses followed established procedures (Ghirardo et al., 2012, 2016). The GC-MS parameters followed those given in Weikl et al. (2016) with the following exceptions: the VOCs were desorbed from twisters by changing the temperatures from 37 to 270°C at the rate of 280°C min^-1^ and holding for 2 min. Before chromatographic separation, samples were cryofocused in the trap of the injection system filled with Tenax TA (Gerstel) at -50°C, following by flash-heating the trap to 270°C at 12°C s^-1^ and holding for 2 min. The GC temperature program was: 40°C for 0 min followed by ramping at 10°C min^-1^ to 130°C and hold for 5 min, then 80°C min^-1^ to 175°C, 2°C min^-1^ to 200°C, 4°C min^-1^ to 220°C, 100°C min^-1^ to 300°C and hold for 6 min. Annotation was performed by comparison of the mass spectra against libraries of reference spectra (NIST 11, Wiley 275) and non-isothermal Kovats retention indices found in literature. Quantification was achieved using response factors calculated using the standards sabinene and α-pinene for MT, linalool for oMT, β-caryophyllene and α-humulene for SQT and geraniol and bornylacetate for oSQT. Other VOCs (oVOCs) were quantified following Kreuzwieser et al. (2014).

### Statistics

For the visualization of the VOC patterns, heat map clusters were calculated using an R program (R Core Team, 2013) and the dendextend package (1.0.1) (Galili, 2015). Random forest analysis was performed using the package “randomForest” (Breiman, 2001) and network analysis using the package “qgraph” (Epskamp et al., 2012) (Fruchterman-Reingold algorithm was applied, *p* < 0.05). Principal component analysis (PCA) of VOCs was performed on SIMCA-P (SIMCA-P v13, Umetrics, Umeå, Sweden). Data was Hellinger transformed to meet the assumption PCA algorithm (Legendre and Legendre, 2012: Müller et al., 2013). First two important principle components were plotted. The bar plot of overall VOC emissions of all treatments and growth inhibition was created by OriginPro 9.0 (OriginLab, Northampton, MA, United States). Significance of growth inhibition was tested by a one-way ANOVA using SPSS (IBM SPSS Statistics 19.0, Duncan’s test, *p* < 0.05). The mycelium areas of the fungi were measured using ImageJ software^1^. Evolutionary analyses of fungi were conducted in MEGA7 (Kumar et al., 2016). The evolutionary history was inferred using the Neighbor-Joining method (Saitou and Nei, 1987). The optimal tree with the branch length sum = 0.78308993 is shown. The tree is drawn to scale, with branch lengths in the same units as those of the evolutionary distances used to infer the phylogenetic tree. The evolutionary distances were computed using the Maximum Composite Likelihood method (Tamura et al., 2004) and are in the units of the number of base substitutions per site. The analysis involved 4 nucleotide sequences. All positions containing gaps and missing data were eliminated. There were a total of 455 positions in the final dataset. Data are shown as mean of 6 ± SEM.

## Results

### Unique Behavior of Selected *Trichoderma* Species in Co-cultivation With *Laccaria bicolor*

**FIGURE 2:**
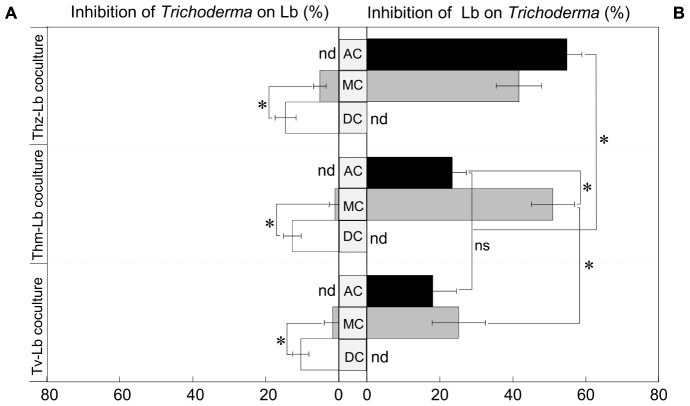
Growth inhibition of **(A)** the three *Trichoderma* species on Lb and **(B)** *vice versa*. Black bar denotes the growth inhibition in air contact (AC) co-cultures, gray bar denotes the growth inhibition in media contact (MC), white bar denotes the growth inhibition in direct contact (DC); nd, not detected. Significances are denoted as asterisks (one-way ANOVA and Duncan’s test, *p* < 0.05), ns, no significance; mean ± SEM, *n* = 6.

To test the performance of the three selected *Trichoderma* species in presence of *L. bicolor* as a non-pathogenic plant mutualistic fungus, we set up a series of direct and indirect confrontation assays (with common head-space) as visualized by representative pictures in Figure 1. Co-cultivations of *Trichoderma* and *L. bicolor* revealed that before direct physical contact on the plates, *Trichoderma* exerted only weak inhibition on *L. bicolor* (1.61–5.12%) (Figure 2A), whereas *L. bicolor* exhibited a much stronger inhibitory effect on all *Trichoderma* species (inhibition rate ranges from 25 to 51%) (Figure 2B). Likewise, also in the co-cultures with only aerial contact (AC), growth of *T. harzianum* was strongly inhibited by *L. bicolor* (54.79 ± 4.15%) with somewhat lower inhibition rates of 23.34 ± 3.89% and 18.02 ± 6.48% for *T. hamatum* and *T. velutinum*, respectively (Figure 2B). The *L. bicolor*-mediated inhibition on *T. hamatum* growth was higher in medium contact (MC) (50.95 ± 5.93%) compared to AC (25.15 ± 7.28%) co-cultures, whereas no differences were found between AC and MC for *T. harzianum* and *T. velutinum* (Figure 2B). When *Trichoderma* and *L. bicolor* got into direct physical contact (DC stage), the colony area of *L. bicolor* was significantly (*p* < 0.05) inhibited by ca. 10.35 ± 2.23% to 14.55 ± 2.88% compared to the MC stage (Figure 2A). By the end of the co-cultivation, *T. harzianum* and *T. hamatum* overgrew *L. bicolor*, whereas *T. velutinum* inhibited *L. bicolor* growth less drastically (Figure 1).

### VOC Emissions From Different *Trichoderma* Species Are Highly Species-Specific

**FIGURE 3:**
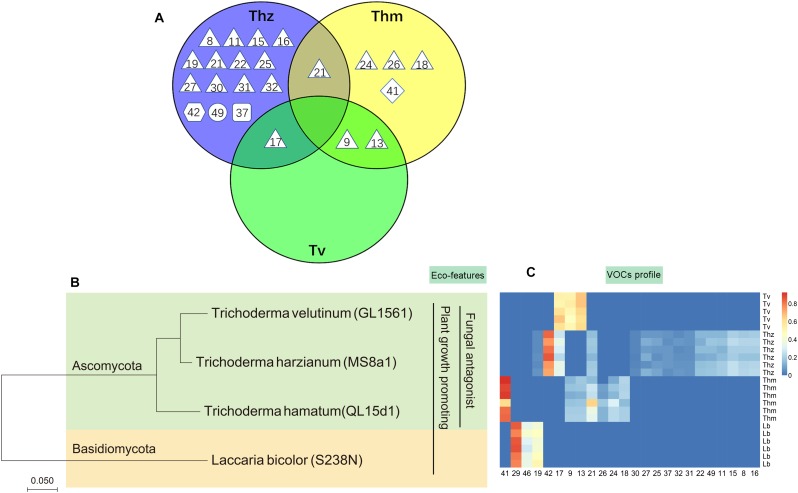
Comparison of volatile organic compound profiles of *T. harzianum* (Thz), *T. hamatum* (Thm), and *T. velutinum* (Tv), and evolutionary relationship of the taxa. **(A)** Venn diagram depicting the VOC profiles of the *Trichoderma* species. Shape of the legend: diamond: monoterpene; hexagon: oxygenated monoterpene; triangle: sesquiterpene, square: oxygenated sesquiterpene; circle: other VOCs. The numbers refer to the compounds listed in Table 1. **(B)** Evolutionary relationships of taxa presented in a phylogenetic tree. The evolutionary history was inferred using the neighbor-joining method (Saitou and Nei, 1987). **(C)** Heat map analysis of the VOCs from the *Trichoderma* species. Data were Hellinger-transformed prior to heat map analysis. Compound numbers refer to Supplementary Table S2.

Overall, 16, 7 and 3 individual VOCs could be detected in the *T. harzianum, T. hamatum*, and *T. velutinum* emission profiles, respectively (Table 1 and Supplementary Table S1). Total emission rate from *T. harzianum* was 68.23 ± 7.68 pmol⋅cm^-2^ h^-1^, whereas *T. hamatum* showed a lower emission intensity of 18.18 ± 1.64 pmol⋅cm^-2^ h^-1^ and *T. velutinum* of 1.60 ± 0.20 pmol⋅cm^-2^ h^-1^ (Table 1). Surprisingly, the three *Trichoderma* species shared no common volatile compound and thus exhibited an extremely species-dependent VOC emission pattern (Figure 3).

**Table 1:** Volatile organic compounds (VOCs) emitted by *T. harzianum* (Thz), *T. hamatum* (Thm), *T. velutinum* (Tv), mean ± SEM, *n* = 6; nd, not detected.

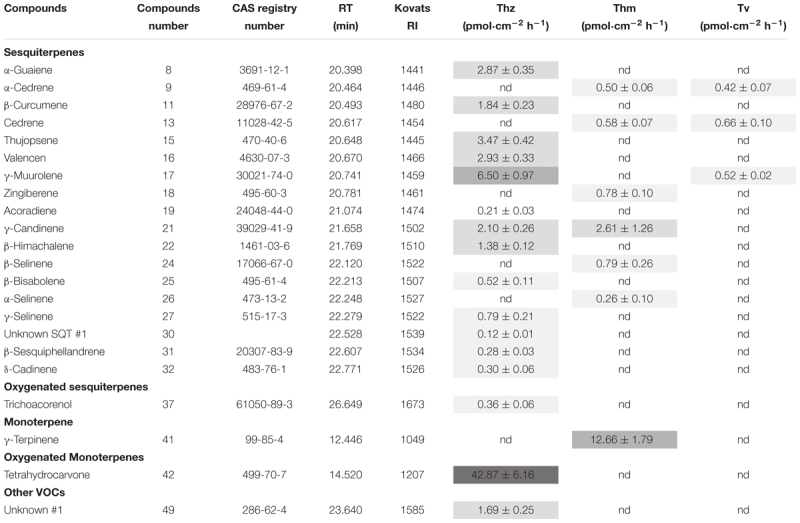


*Colors indicate VOC emission strengths; dark gray: very high (>26 pmol⋅cm^-*2*^ h^-*1*^), medium gray: high (6–13 pmol⋅cm^-*2*^ h^-*1*^), gray: medium (1–5 pmol⋅cm^-*2*^ h^-*1*^), light gray: low (0–1 pmol⋅cm^-*2*^ h^-*1*^).*

The VOCs of *T. harzianum* encompassed 13 SQTs and one major oxygenated sesquiterpene (oSQTs), which contributed to ca. 35% of the total emission. The emission profile of this species was further dominated by the oxygenated monoterpene (oMT) tetrahydrocarvone, which amounted up to 62.05 ± 0.04% of the total emission (Table 1). Of the 16 emitted VOCs from *T. harzianum*, 14 VOCs were unique compared to the two other *Trichoderma* species (Figure 3A). The most abundant compounds of *T. hamatum* were the MT γ-terpinene and the SQT γ-candinene with ca. 70 and 14% of the total emission, respectively. These compounds were not emitted by the two other *Trichoderma* species (Table 1 and Figure 3C).

*Trichoderma velutinum* emitted quantitatively and qualitatively the lowest number of VOCs compared to the two other *Trichoderma* species, and its emission pattern contained no unique compound compared to the other two species (Table 1 and Figure 3A). No significant differences were detected in the fungal area of the three *Trichoderma* species (the mean fungal area being 11.7 ± 0.78 cm^2^), and the VOC emissions were normalized to fungal area. Thus, the large differences in VOC emission we observed here among the *Trichoderma* species are unlikely to derive from species-specific growth performance or related fungal area density.

### Different Contact Scenarios of *Trichoderma* spp. and *Laccaria bicolor* Trigger Changing Emission Profiles

Compared to axenic cultures, different co-cultivation scenarios were found to induce specific changes in the fungal VOC emissions. Already AC between the *Trichoderma* species and *L. bicolor* was sufficient to trigger some changes in the overall emission profile. Four new compounds [α-selinene, limonene, cyclohexane, 1,2,4-tris(methylene)-], were detected in AC co-culture of *T. harzianum* and *L. bicolor*, whereas *T. harzianum*-derived trichoacorenol could no longer be detected (Supplementary Figure S1B). Likewise, when *T. velutinum* and *L. bicolor* were grown together in AC, the two *T. velutinum*-originating SQTs α-cedrene and cedrene as well as (+)-cuparene from *L. bicolor* were now absent. In contrast, one new MT, limonene, was detected (Supplementary Figure S1C). Notably, the same SQT compounds (i.e., α-cedrene and cedrene) disappeared from the emission profile of *T. hamatum* when it was in AC with *L. bicolor*, as well as all compounds from the axenic emission profile of *L. bicolor* itself (Supplementary Figure S1).

Compared to AC, co-culturing in medium contact (MC) could additionally influence the VOC emissions by nutrient depletion and via communication through soluble secondary metabolites. The volatile profiles of the AC and MC co-cultured *Trichoderma*–*L. bicolor* mycelia were, however, nearly the same: As a new compound, the MT limonene was found in the emission profile of AC-cultured *T. harzianum* and AC-cultured *T. velutinum*, whereas this compound was not detected in MC cultivation. The two SQTs α-cedrene and cedrene originally emitted by *T. velutinum* and *T. hamatum* alone, where neither observed during AC nor during MC co-cultivation.

**FIGURE 4:**
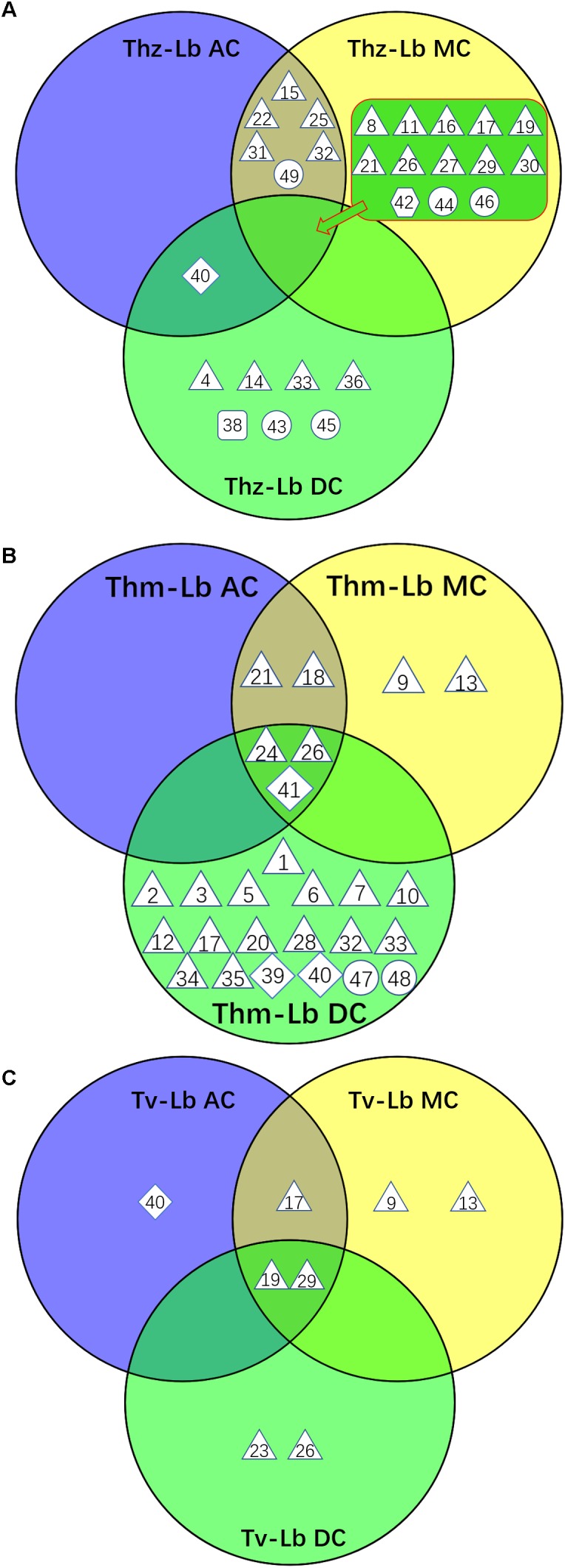
Venn diagram depicting the VOC profiles from the co-cultures of three *Trichoderma* species with *L. bicolor*. **(A)** VOCs from three *T. harzianum* – *L. bicolor* co-cultures, **(B)** VOCs from three *T. hamatum* – *L. bicolor* co-cultures, **(C)** VOCs from three *T. velutinum* – *L. bicolor* co-cultures. Shape legend: diamond: monoterpene; hexagon; oxygenated monoterpene; triangle: sesquiterpene, square: oxygenated sesquiterpene; circle: other VOCs. The numbers refer to the compounds listed in Supplementary Table S2.

**FIGURE 5:**
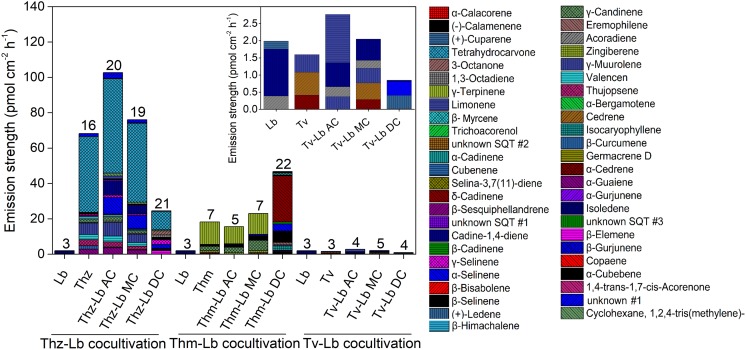
The volatile organic compound profiles of *T. harzianum* (Thz), *T. hamatum* (Thm), *T. velutinum* (Tv), and *L. bicolor* (Lb) alone and each *Trichoderma* strain in different co-cultivation set-ups with Lb. For further information on the abbreviations please see Figure 1. The numbers on the bars indicate the total individual VOCs detected. Insert: Magnification of the VOC profile of Tv and sole Lb culture. Emission rates of each compound calculated based on the fungal area and collection duration of VOCs; values are means of 6 replicates.

Direct contact between the fungi may induce specific communication- and defense-related signals within the fungi, which might also manifest themselves in the fungal emissions. Thus, we further compared the direct physical contact (DC) emission profiles to the AC and MC scenarios (Figure 4, 5). Considering the emission rates, the *Trichoderma* species behaved contrary to each other. For *T. harzianum*, a dramatic decrease for all VOC concentrations was measured in DC with *L. bicolor*, except for γ-selinene, which increased notably (Figure 5). Indeed, almost all the common compounds between AC and MC pattern had a higher emission rate in AC than in MC, and almost all the compounds common between DC and MC showed a higher emission rate in MC than in DC in the *T. harzianum–L. bicolor* co-culture. Though the emission intensity in *T. harzianum*–*L. bicolor* co-cultivation decreased and was only around one fourth of that measured in AC co-cultures, 21 compounds were detected in DC (Figure 5); 7 of them being new and unique for the DC confrontation scenario (Figure 4). Compared to axenic cultures of the individual fungi, a drastic change in *T. harzianum*–*L. bicolor* VOC profile was observed: emissions of 7 compounds originally detected from *T. harzianum* were absent (thujopsene, β-himachalene, β-bisabolene, β-sesquiphellandrene, δ-cadinene and unknown #1), while 10 new compounds were detected [β-elemene, α-bergamotene, α -selinene, selina-3,7(11)-diene, unknown SQT #2, 1,4-*trans*-1,7-*cis*-acorenone, limonene, 1,3-octadiene, 3-octanone, cyclohexane,1,2,4-tris(methylene)-] (Supplementary Figure S1A).

In contrast to *T. harzianum*, the overall *T. hamatum* emission rate notably increased in DC contact compared to other co-cultivation scenarios. By far the highest compound diversity was, moreover, detected in DC between both mycelia (22 compounds) compared to AC or MC (5 and 7 compounds, respectively) (Figure 4, 5). Of the 22 compounds detected in DC, 19 were new, while 4 of the 5 compounds originally detected in AC cultures were absent (Supplementary Figure S1A). Compared to MC cultures, three SQTs (β-selinene, α-selinene, and δ-cadinene) showed ca. 2.5-, 5-, and 26-fold concentration increase, respectively, in DC co-cultivation. In contrast, the MT γ-terpinene decreased from 11.79 pmol⋅cm^-2^ h^-1^ in MC to almost zero in DC co-cultivation.

In the context of *T. velutinum* and *L. bicolor* co-culture, 2 out of 5 detected SQTs [α-selinene and (+)-ledene] were specific for the DC culture. Moreover, three SQTs (α-cedrene, cedrene, and γ-muurolene) originally from *T. velutinum* were absent, and the emission of 2 compounds (acoradiene, and cadine-1,4-diene) (Figure 5 and Supplementary Figure S1C) was strongly decreased.

### VOC-Based Characterization of Axenic and Co-cultured *Trichoderma* Species and *Laccaria bicolor*

**FIGURE 6:**
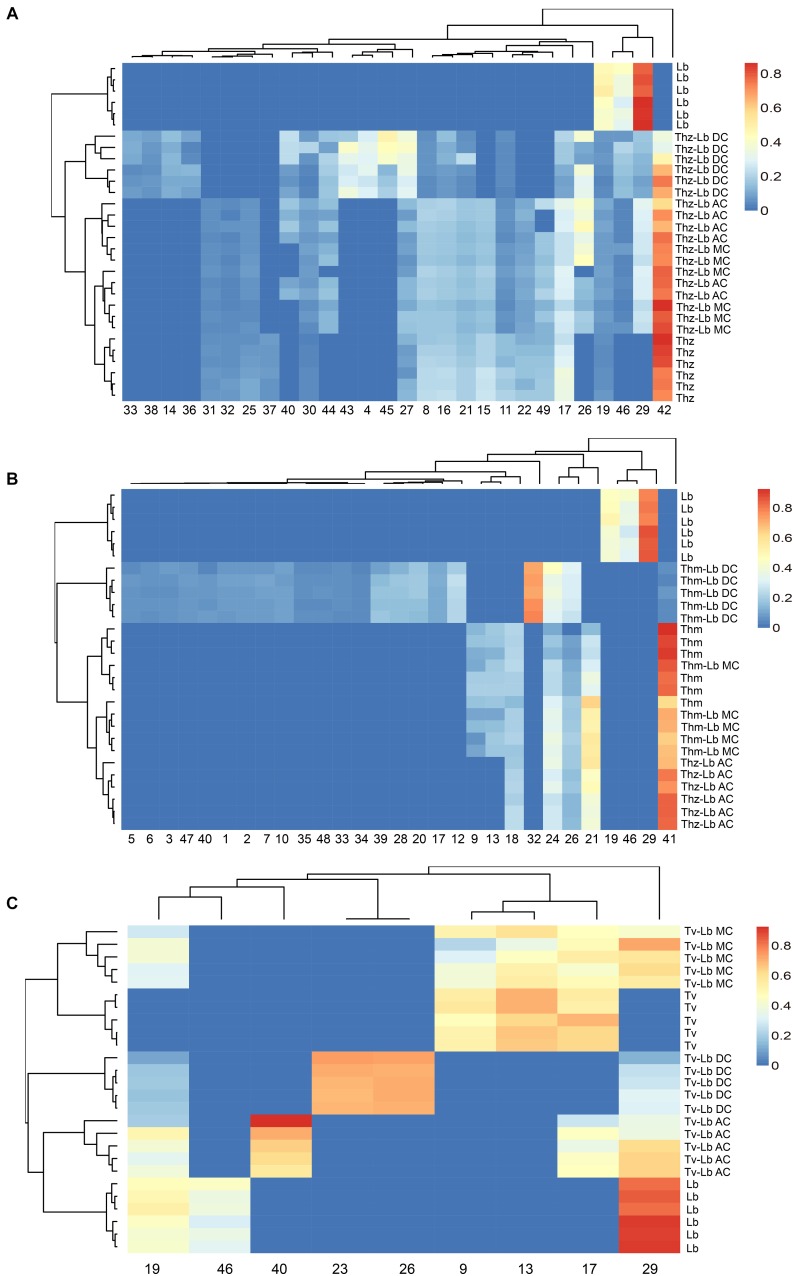
Heat map clustering of VOC profiles from *T. harzianum* (Thz), *T. hamatum* (Thm), *T. velutinum* (Tv), *L. bicolor* (Lb), and *Trichoderma*- Lb co-cultures. **(A)** VOC profiles of Thz, Lb and their co-cultures; **(B)** VOC profiles of Thm, Lb and their co-cultures; **(C)** VOC profiles of Tv, Lb and their co-cultures. Data were Hellinger-transformed prior to clustering. NC, no contact; AC, airborne contact; MC, media contact; DC, direct contact. Compound numbers refer to Supplementary Table S2.

**FIGURE 7:**
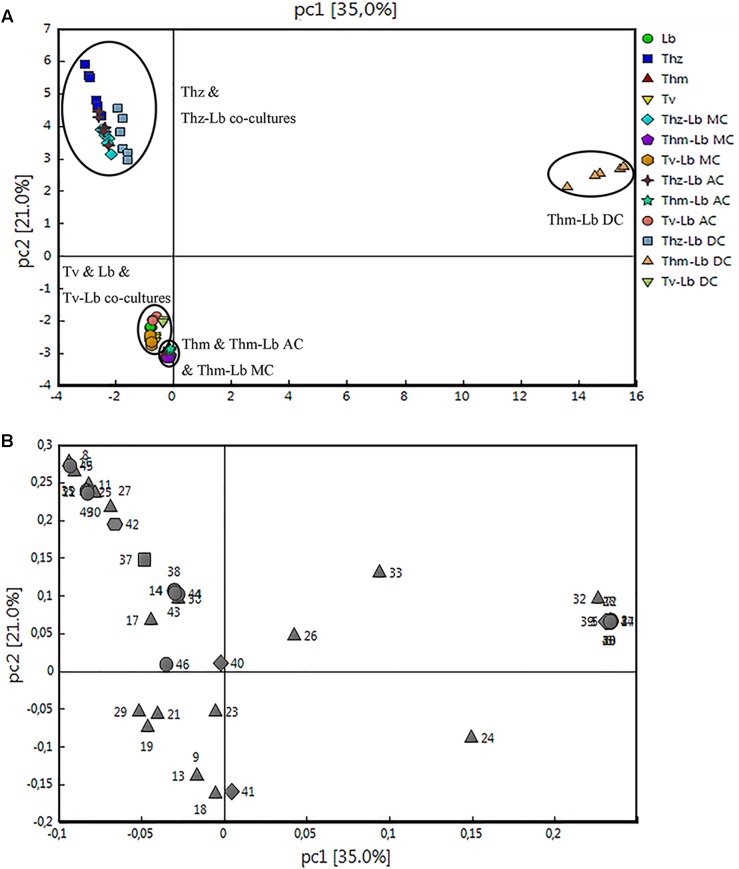
Principal component analysis of the volatile organic compound emission patterns released from *T. harzianum* (Thz), *T. hamatum* (Thm), and *T. velutinum* (Tv) co-cultivated with *L. bicolor* (Lb). The scores **(A)** of the first two principal components (PC 1 and PC2) for the different species and co-cultivations are presented with the loadings **(B)**. The detected compounds were divided into five different chemical groups (loading plot): diamond, monoterpene; hexagon, oxygenated monoterpene; triangle, sesquiterpene; square, oxygenated sesquiterpenes; circle, other VOCs.

Hierarchical clustering (heat maps in Figure 6A–C) and a principal component analysis (PCA; Figure 7A,B) revealed a clear separation of the VOC profiles of individual *Trichoderma* species, *L. bicolor* and their interactions. PCA, for example, highlighted particularly the differences between the *Trichoderma* species (Figure 7A), while the heat maps clearly show that also the interactions of *Trichoderma* spp. with *L. bicolor* resulted in unique emission patterns, making it possible to separate the different confrontation scenarios according to the VOC profiles (Figure 6A–C). For *T. velutinum* and *T. hamatum*, the emission patterns were specific for each co-culture (AC, MC, and DC; Figure 6B,C) with the *T. hamatum*–*L. bicolor* DC condition clearly diverging the most from the others (see also separate cluster in PCA in Figure 7A). Also *T. harzianum* and *L. bicolor* DC co-culture was separated from the others, although a clear separation could not be detected between AC or MC (Figure 6A). Random forest analysis of the VOC emissions proposes a relative contribution of different compounds for separating groups (Supplementary Figure S2). In the co-cultivation of *T. harzianum* and *L. bicolor*, the MT limonene was the most important volatile accounting for the separation, whereas the same compound was rather inconsequential in the *T. hamatum*–*L. bicolor* interaction. The SQT cadine-1,4-diene, on the other hand, explained much of the differentiation in both *T. harzianum*–*L. bicolor* and *T. velutinum*–*L. bicolor* interactions (Supplementary Figure S2).

The correlations of individual compounds are visualized in a correlation network (Supplementary Figure S3). In the interaction of *T. harzianum* and *L. bicolor*, all the compounds that were specific for direct physical contact displayed a positive correlation (*p* < 0.05). Most of the compounds shared by AC and MC co-cultures correlated positively as well, except for the SQTs γ-selinene and (+)-cuparene, that showed negative correlation. The compounds detected in the DC co-cultivation were negatively correlated with the compounds detected in AC and MC co-cultures. Only the SQTs γ-selinene and (+)-cuparene did not follow this pattern (Supplementary Figure S3A).

In the *T. hamatum*–*L. bicolor* co-cultivation, the compounds showed three groups of positive correlation (*p* < 0.05) (compounds from *L. bicolor*, compounds shared with AC and MC co-culture, and compounds specific for DC co-culture). However, these three clusters of compounds were negatively correlated to each other. The two SQTs β-selinene and α-selinene, which were detected in both, AC and MC, co-cultures, showed a positive correlation with DC-specific compounds but negative correlation with compounds in their own group (Supplementary Figure S3B). In the co-cultivations of *T. velutinum* and *L. bicolor*, the compounds also correlated in three groups (compounds from *L. bicolor*, compounds specific for MC co-culture and compounds specific for DC co-culture). In between each of these groups, the correlation was negative in all cases (Supplementary Figure S3C).

## Discussion

Considering the enormous number of *Trichoderma* species and strains known to date, the VOC profiles of only a very limited number of fungi have been explored so far. We investigated the VOC emissions of three *Trichoderma* species, of which *T. harzianum* was the strongest emitter, followed by *T. hamatum* and *T. velutinum*. The VOC profiles of the three species were highly species-dependent and dominated by SQTs. With respect to *T. harzianum*, the abundance of SQTs was surprisingly high, as thus far only a few SQTs have been reported from this species (Lee et al., 2016; Li et al., 2018). Previous studies have shown, though, that the VOC profiles of different *T. harzianum* strains can vary tremendously (Nemcovic et al., 2008; Siddiquee et al., 2012; Lee et al., 2016; Nieto-Jacobo et al., 2017). In the study of Lee et al. (2016), *T. harzianum* CBS 227.95 emitted 27 VOCs, whereas *T. harzianum* CBS 226.95 only emitted 3 VOCs. Astonishingly, Siddiquee et al. (2012) detected even 278 VOCs in the *T. harzianum* strain FA1132. Apart from the genetic differences between strains, the variations in VOC patterns could also be due to discrepancies in nutrient availability (Nieto-Jacobo et al., 2017; Gonzáles-Pérez et al., 2018), developmental stage of the fungi (Weikl et al., 2016), and technique applied during VOC collection and analysis. In the present study, Melin-Norkrans synthetic growth medium was chosen as nutrient source based on previous experience. Müller et al. (2013), who screened several soil fungi, including *Trichoderma* spp. and *L. bicolor*, for their volatiles initially chose this medium based mainly on two criteria: (1) to avoid high background volatiles that could disturb the VOC analyses, and (2) to use a common medium for all tested strains for better comparability. The use of a common medium, despite being sub-optimal for some strains (Supplementary Figure S4), is essential when aiming to compare the VOC profiles across species (Polizzi et al., 2012). Nonetheless, if other non-chemotaxonomic aims are of interest, also screening of fungi under varying environmental conditions, including different media and in contact with other organisms, seems to be essential to reveal the whole VOC emission potential of fungal species.

So far, only scattered information exists on the VOCs of *T. hamatum*, and the VOC profiles of *T. velutinum* are completely uninvestigated. *T. velutinum* is a relatively new taxon of *Trichoderma* identified only in 2003 (Bissett et al., 2003). While this species has been reported to have good biocontrol potential (Matarese et al., 2012; Sharma et al., 2017), our results identified *T. velutinum* as a comparably low SQT emitter. Only few (3) SQTs at low emission rates were detected from axenic cultures of *T. velutinum*. For *T. hamatum*, 17 different VOCs have been previously reported (Siddiquee, 2014). In the present study, we detected seven VOCs composed of six SQTs and one MT, none of which having been reported previously from *T. hamatum*. Altogether therefore, 24 compounds are now reported from axenic cultures of *T. hamatum*. Notably, testing different strains of one species thus also appears to be a promising strategy to identify a more complete set of secondary metabolites this species is able to generate and thus to potentially activate otherwise silent genes or gene clusters.

Volatile organic compounds, and in particular SQTs, are able to quickly move through pores in the soil matrix and thus have the potential to mediate belowground, long-distance chemical signaling (Penuelas et al., 2014). Accordingly, several studies suggest that microbial VOCs are not a metabolic waste, but important signaling compounds in the soil (Schmidt et al., 2016, 2017; Weisskopf et al., 2016). Due to the biocontrol properties of *Trichoderma* spp., the functions of VOCs in *Trichoderma* interactions have been tested mostly in the context of controlling fungal pathogens of plants. Provided that *Trichoderma* spp. are antagonistic also against plant-beneficial fungi or other beneficial organisms in the soil, the benefit of commercial applications of *Trichoderma* spp. in forestry or agricultural practice might vary depending on the microbial communities already present at the site. Our study revealed that the outcome of the interaction between the three studied *Trichoderma* species and the ectomycorrhizal fungus *L. bicolor* depends strongly on the degree and type of contact. Surprisingly, we found that over longer distances, *L. bicolor* exerted more negative impact on the growth of *Trichoderma* than *vice versa*. As the growth inhibition of *Laccaria* on *T. harzianum* or *T. velutinum* did not differ between AC and MC cultivation, these results indicate that VOCs are important effectors in inhibiting *Trichoderma* growth over longer distances via airborne signaling. One or several of the detected three SQTs [acoradiene, cadine-1,4-diene and/or (+)-cuparene] might therefore be biologically active, even though also other undetected compounds might be involved. As the growth of *Laccaria* was only marginally affected at the same time, the VOCs emitted by *Trichoderma* at that stage probably have no or only a weak role in the antagonism against *L. bicolor*. The fact that *L. bicolor* deployed stronger inhibition on *T. hamatum* in MC compared to AC culture suggests the additional involvement of soluble compounds as effectors toward *T. hamatum*. However, the observed growth inhibition might also be due to competition of important nutrients. Interestingly, DC between *Trichoderma* species and *L. bicolor* resulted in an opposing outcome, as all the *Trichoderma* species showed antagonism to *L. bicolor* in this scenario. *T. hamatum* and *T. harzianum* visibly overgrew *L. bicolor*, whereas the antagonism of *T. velutinum* was milder. These observations indicate that *Trichoderma* antagonism is most effective at close distance, probably by involving enzyme-coupled attacks (such as by proteases and chitinases).

In addition to the differences in antagonism toward each other, the co-cultivation scenarios also exhibited changing VOC profiles. In the case of *T. hamatum*, the VOC emission increased with increasing contact degree with *L. bicolor*. Not only the emission rate but also the diversity of VOCs in the emission blend was enhanced in DC compared to the situation when the fungi were without physical contact. Previously, it had been shown that fungal and microbial VOC emissions strongly depend on the abiotic and biotic environment (Schmidt et al., 2016, 2017). Microbial genomes possess large sets of functionally unknown genes that may be expressed only under specific environmental conditions (Zhou et al., 2004). For example, Schmidt et al. (2017) observed a strongly altered metabolism of the bacterium *Serratia plymuthica* when in contact with the VOCs from the fungal pathogen *Fusarium culmorum*. The rare terpene compound sodorifen, for instance, has been only detected form *S. plymuthica* when exposed to *Fusarium* volatiles (Schmidt et al., 2017). So far, only few studies addressed the VOCs in co-cultivation of two fungi. Weikl et al. (2016) investigated the VOCs of two *in vitro* co-cultured plant pathogenic fungi, *Alternaria alternata* and *Fusarium oxysporum*. Similar to *T. hamatum* in our study, the authors showed an increase of total VOC emissions when the two fungi were in DC. In contrast to Weikl et al. (2016), however, our results revealed that the VOC profiles completely changed in DC compared to other confrontation scenarios. For example, the MT γ-terpinene dominated the VOC profile of *T. hamatum* in all measurements except in DC, in which the compound accounted only for 0.3% of the total VOCs, while the SQT δ-cadinene was now the prevalent VOC. δ-cadinene synthase (CDNS) has been previously studied in *Gossypium barbadense* plants in which the CDNS activity and gene expression correlated with the resistance against cotton pathogens (Bianchini et al., 1999; Townsend et al., 2005). However, whether δ-cadinene may have a role in *Trichoderma* defense needs to be investigated in more detail. Similar to δ-cadinene, also isocaryophyllene was detected only when *T. hamatum* and *L. bicolor* grew in DC. Isocaryophyllene is an isomer of caryophyllene, whose microbial emission was previously shown to induce growth of lettuce (Minerdi et al., 2011).

In contrast to *T. hamatum*, more physical contact between *T. harzianum* or *T. velutinum* and *L. bicolor* decreased the overall VOC release. Nevertheless, interesting changes in the VOC profiles of both of these *Trichoderma* species in DC with *L. bicolor* appeared when compared to other confrontation scenarios. For example, the synthesis of tetrahydrocarvone, which dominated the emission profile of axenic *T. harzianum* cultures, was probably suppressed, as its emission decreased to only one fourth. Tetrahydrocarvone is an oxygenated MT and is to our knowledge reported for the first time from fungi. Also the emission of thujopsene, a SQT which was previously shown to induce lateral root growth in *Arabidopsis* and poplar (Ditengou et al., 2015), was suppressed in DC compared to all other tested growth conditions. In addition to the diminished compounds, DC between *T. harzianum* and *L. bicolor* also induced the emission of many new compounds, such as β-elemene, α-selinene, 1,3-octadiene and 3-octanone. Exposure to 3-octanone was previously shown to induce resistance in *Arabidopsis* against pathogenic bacteria (Naznin et al., 2014). In general, one possible explanation for the lower emission rates might be that the respective fungi rather invest in soluble than volatile compounds upon close-range (physical) contact. Also, nutrient competition between *Trichoderma* and *L. bicolor* or uptake and degradation of volatiles by one or both of the fungi might cause a decrease in the apparent VOC emission rates.

Regarding *L. bicolor* emission profiles, interesting discrepancies between different contact degrees were observed: the emission of (+)-cuparene, a SQT that has been previously shown to possess antimicrobial activity against fungi and bacteria (Ishikawa et al., 2001), was up-regulated when grown in DC with *T. harzianum.* This SQT, however, completely disappeared when *L. bicolor* was encountered by *T. hamatum* or *T. velutinum*, suggesting a species-specific response of *L. bicolor*. Alternatively, consumption of the (+)-cuparene by *T. hamatum* and *T. velutinum* is also possible. In *L. bicolor*, the emission of two other SQTs, acoradiene and cadine-1,4-diene were, moreover, significantly down-regulated in all direct confrontation scenarios compared to axenic cultures of *Laccaria*. However, whether these compounds are responsible for repressing *Trichoderma* growth in AC contact remains to be elucidated.

Overall, the present study demonstrates that *Trichoderma* species do not only differ in their emission profiles, but that the volatile emissions are also strongly adjusted according to the biotic environment. Previously Weikl et al. (2016) also showed altered VOC profiles when two fungi, *A. alternata* and *F. oxysporum*, grew in different contact degrees with each other. Interestingly, in the present study the strongest emission rates of single volatiles were measured when two beneficial species had highest distance to each other, suggesting ecological importance of VOCs in long-range interactions of plant-beneficial fungi. In particular, *L. bicolor*-emitted VOCs appear to be involved in the repression of *Trichoderma* before direct physical contact. Our results therefore verify the notion that fungi are able to regulate their VOC emissions according to the environmental constraints, supporting the hypothesis that fungal VOCs have important ecological functions in microbial interactions.

Multivariate analysis of our results furthermore revealed that different *Trichoderma* species possess individual VOC patterns, potentially allowing the utilization of VOCs as biomarkers for the identification of fungi (Neerincx et al., 2016). However, our study also demonstrated the complexity and adjustability of the fungal secondary metabolism in different environmental conditions. While this allowed us to differentiate between the three different co-cultivation scenarios, these dynamics also question the general suitability of VOCs as biomarkers in different labs and from different samples etc. Considering the enormous amount of different *Trichoderma* species (Bissett et al., 2015) as further challenge, a lot of efforts are still needed to understand the ecological function of VOCs from various *Trichoderma* strains and species.

The presented results reveal new aspects on possible functions of the *Trichoderma* genus used as biocontrol agent. The antagonistic nature of the here tested *Trichoderma* is apparently not limited to plant pathogens but may also affect plant-beneficial fungi. Previous studies on beneficial effects of co-inoculation of crop plants with arbuscular mycorrhizal (AM) fungi and *Trichoderma* spp. suggest varying compatibilities of different beneficial microbial species. For example, *T. atroviride* showed mycoparasitic behavior toward *Gigaspora* spp. when co-inoculated on *Medicago truncatula* plants (Lace et al., 2015). Similarly, Lagos et al. (2018) observed a strong mutual inhibition between *Rhizophagus irregularis* and *Trichoderma viride*. In contrast, in the study of Colla et al. (2015), *R. irregularis* co-inoculated with *T. atroviride* enhanced crop growth more than each of the microorganisms alone. Also Kabdwal et al. (2019) observed best plant performance when multiple plant-beneficial microorganisms, including *T. harzianum* and AM fungi, were applied. Since soil-born beneficial microbes play a pivotal role in the functioning of plants by influencing their physiology and development (Mendes et al., 2013), further studies are needed to investigate the possible outcome when a plant is involved in interactions between different beneficial agents, including ectomycorrhizal fungi. Clearly, the beneficial microbiome of the rhizosphere might be affected due to application of commercial biocontrol species (Schulz-Bohm et al., 2018). In our study, *T. harzianum* and *T. hamatum* showed antagonistic behavior against *L. bicolor* in DC, whereas the low VOC emitter *T. velutinum* was least antagonistic against the mycorrhizal fungus. Still, *T. velutinum* was already shown to be very effective against several plant-pathogenic microbes (Matarese et al., 2012; Sharma et al., 2017). Should it be able to grow concomitantly with other plant beneficial fungi, the use of this species might be highly advantageous in biocontrol. These results suggest that by choosing the right *Trichoderma* species for biocontrol purposes, agriculture and forest management could be further optimized.

## Author Contributions

YG, JB, J-PS, and MR designed the study. YG performed the experiments and analyzed the data. YG coordinated cultivation and VOC sampling. YG performed GC-MS analysis together with AG and BW. YG and MR wrote the first draft of the manuscript and prepared the figures. All authors contributed to data analysis, interpretation of the findings, and edited and approved the manuscript.

## Conflict of Interest Statement

The authors declare that the research was conducted in the absence of any commercial or financial relationships that could be construed as a potential conflict of interest.

## Footnotes

1 http://rsbweb.nih.gov/ij/download.html

## Abbreviations

AC: airborne contact
DC: direct contact
MC: media contact
MT: monoterpene
SQT: sesquiterpene
VOCs: volatile organic compounds
