# Supplementary material for: Trichoderma Species Differ in Their Volatile Profiles and in Antagonism Toward Ectomycorrhiza Laccaria bicolor
Source: Front Microbiol. 2019 Apr 26;10:891. doi: 10.3389/fmicb.2019.00891 (PMC6499108; doi:10.3389/fmicb.2019.00891)
Supplement: Supplementary file 1 [file Data_Sheet_1.pdf]

Supplementary Figures

Supplementary Figure S1. Venn diagrams depicting the VOC profiles of *L. bicolor* (Lb), *T. harzianum* (Thz) and Lb-Thz co-cultures **(A)**; Lb, *T. hamatum* (Thm) and Lb-Thm co-cultures **(B)**; Lb, *T. velutinum* (Tv) and Lb-Tv co-cultures **(C)**. Shape legend: Diamond: monoterpene; hexagon; oxygenated monoterpene; triangle: sesquiterpene, square: oxygenated sesquiterpene; circle: other VOC. The numbers refer to the compounds listed in Supplementary Table S2.

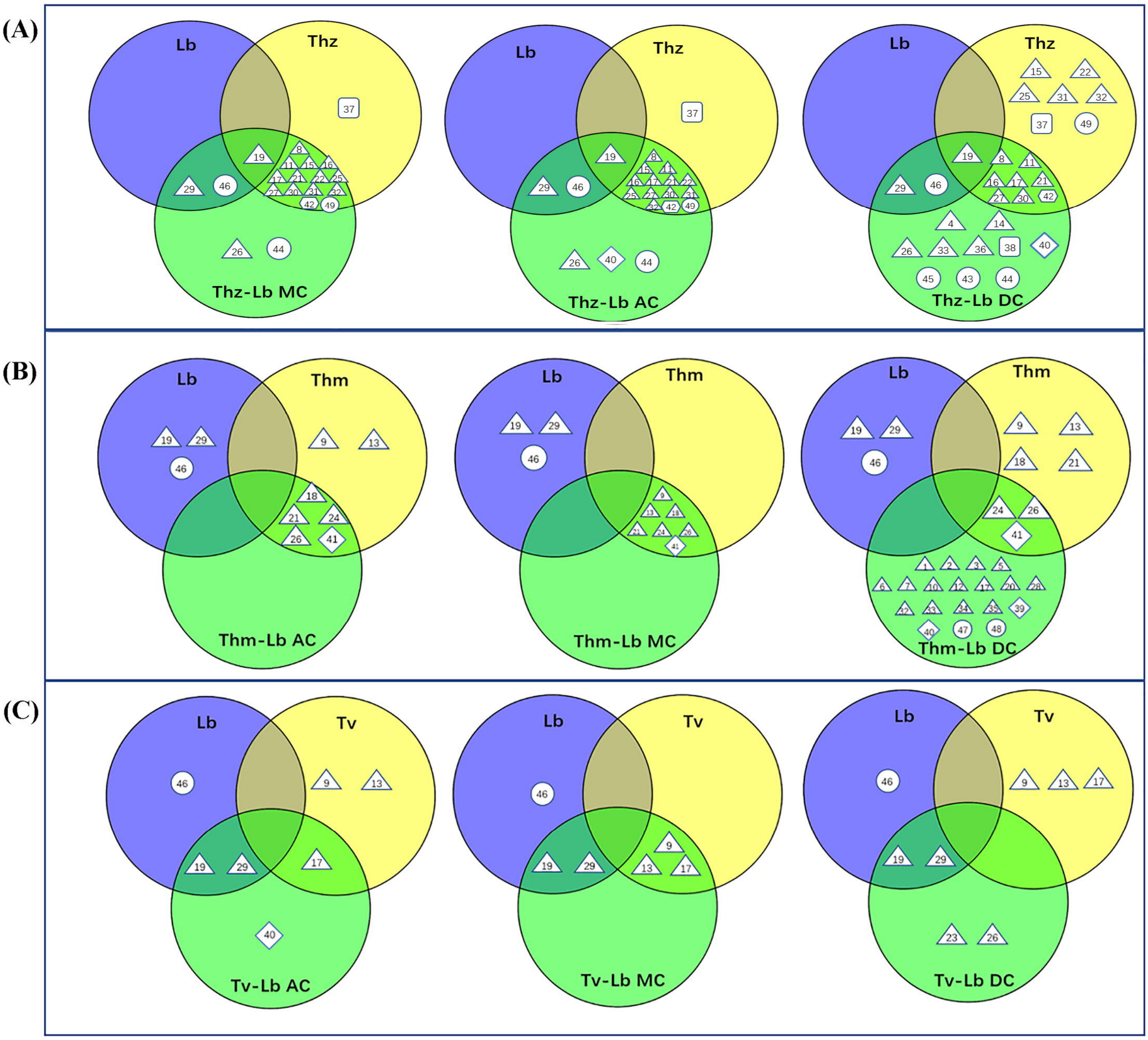

Supplementary Figure S2. Random forest analyses (Breiman, 2001) of VOC profiles of *L. bicolor* (Lb), *T. harzianum* (Thz), and Lb-Thz co-cultures **(A)**; Lb, *T. hamatum* (Thm), and Lb-Thm co-cultures **(B)**; Lb, *T. velutinum* (Tv), and Lb-Tv co-cultures **(C)**. Random forest analysis consists of a supervised classification technique based on an ensemble of decision trees that are able to identify the important variables. In the current analysis, classification model was applied. The number of trees to grow (ntree) was set to 500 to get the lowest error rate: The number of predictors sampled for splitting at each node (mtry) was set to default. The present random forest analysis is based on the total VOCs from three *Trichoderma*-driven interactions. The y-axis represents the compounds in order of importance in each co-culture, from top to bottom. The confusion matrixes show the prediction accuracy based on the random forest result (actual against predicted group).

(A)

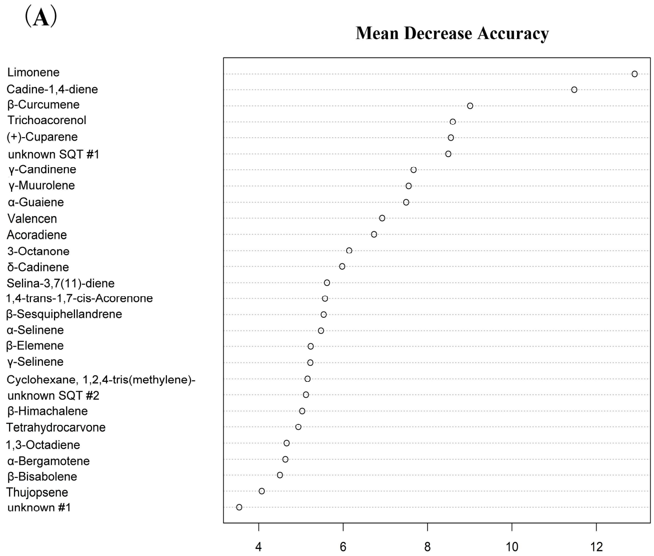

	Predicated group					
Confusion matrix	Lb	Thz	Thz-Lb AC	Thz-Lb DC	Thz-Lb MC	Class error
Lb	6	0	0	0	0	0
Thz	0	6	0	0	0	0
Thz-Lb AC	0	0	6	0	0	0
Thz-Lb DC	0	0	0	6	0	0
Thz-Lb MC	0	0	2	0	4	0.33
Prediction accuracy	100%	100%	75%	100%	67%	

Actual group

(B)

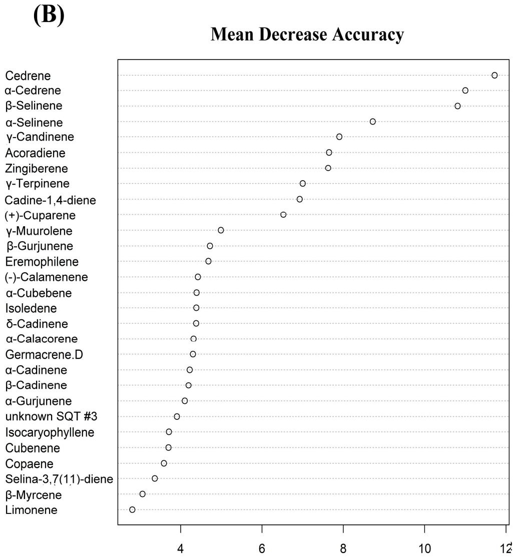

	Predicated group					
Confusion matrix	Lb	Thm	Thm-Lb AC	Thm-Lb DC	Thm-Lb MC	Class error
Lb	6	0	0	0	0	0
Thm	0	5	0	0	1	0
Thm-Lb AC	0	0	6	0	0	0
Thm-Lb DC	0	0	0	5	0	0
Thm-Lb MC	0	2	1	0	2	0.6
Prediction accuracy	100%	71%	86%	100%	40%	

Actual group

(C)

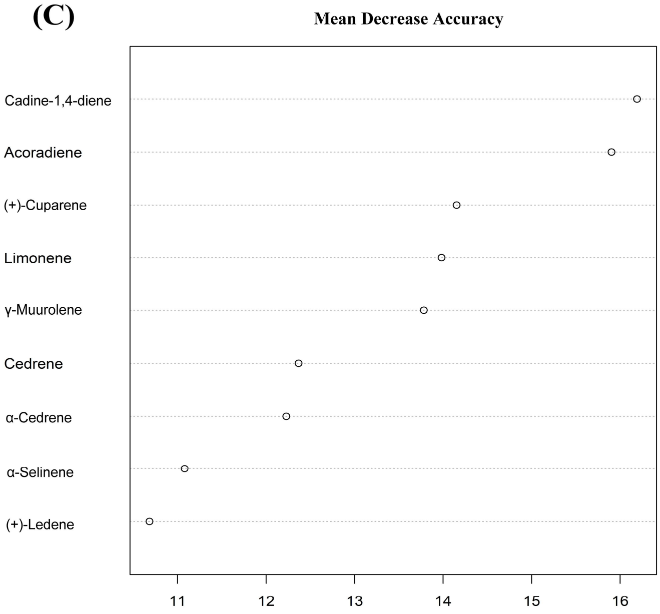

	Predicated group					
Confusion matrix	Lb	Tv	Tv-Lb AC	Tv-Lb DC	Tv-Lb MC	Class error
Lb	6	0	0	0	0	0
Tv	0	5	0	0	0	0
Tv-Lb AC	0	0	5	0	0	0
Tv-Lb DC	0	0	0	5	0	0
Tv-Lb MC	0	0	0	0	5	0
Prediction accuracy	100%	100%	100%	100%	100%	

Actual group

Supplementary Figure S3. Network visualization of correlations of VOCs detected in each *Trichoderma*-driven interaction. **(A)** Correlations of VOCs from *T. harzianum-L. bicolor* co-culture; **(B)** correlations of VOCs from *T. hamatum-L. bicolor* co-culture; **(C)** correlations of VOCs from *T. velutinum-L. bicolor* co-culture. Nodes denote the compounds and compound numbers refer to Supplementary Table S2. Edges denote the correlations between each compound, for which green edges indicate positive correlations and red edges indicate negative correlations ($p < 0.05$). The color saturation and the width of the edges corresponds to the absolute weight and scale relative to the strongest weight in the graph; i.e. the higher the correlation, the thicker and more saturated is the edge. The letter "X" before compound numbers was used to differentiate numerical variables in R.

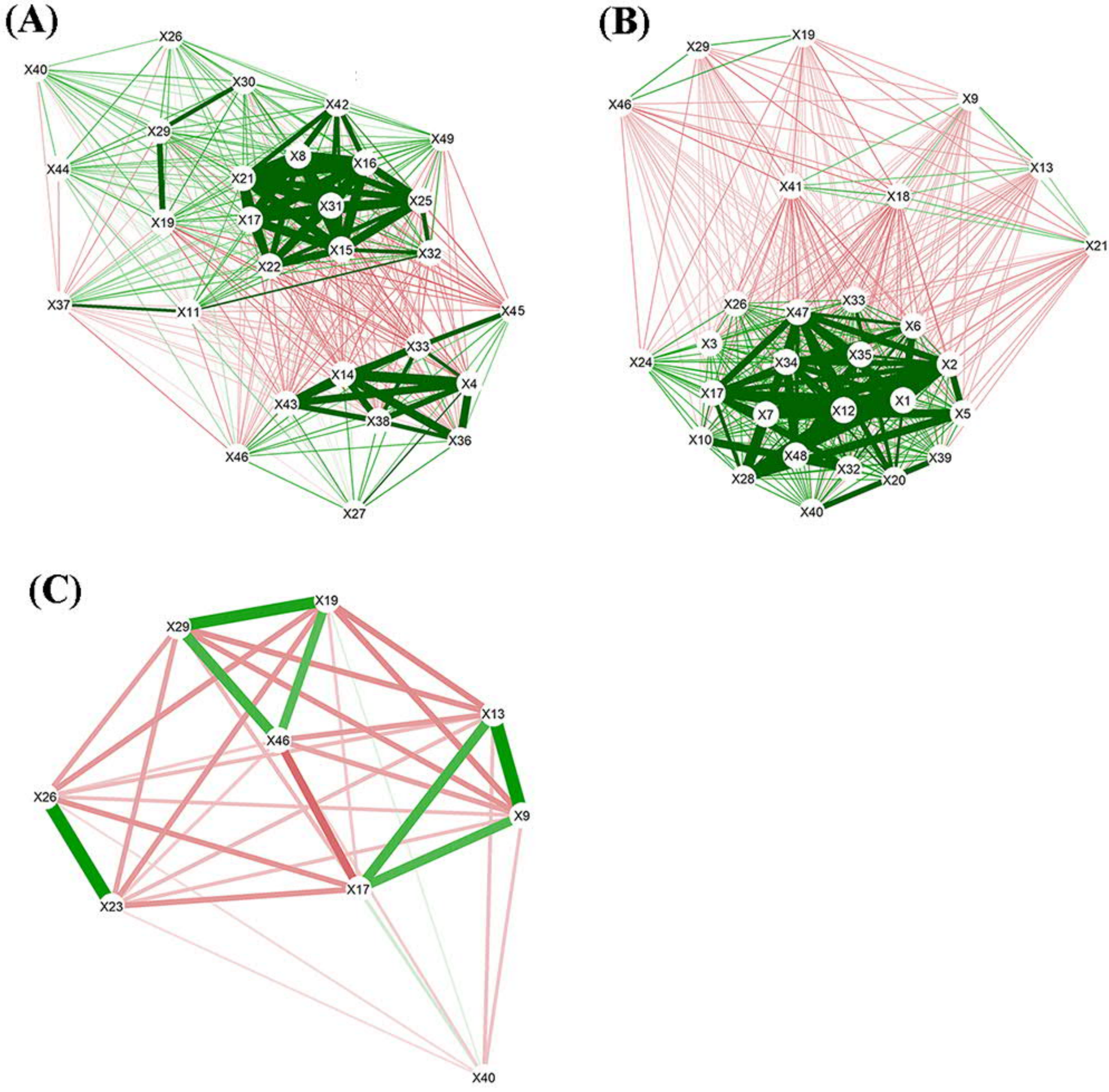

Supplementary Figure S4 The growth rate (**A, C, E**) and culture morphology (**B, D, F**) of *T. harzianum*; (**A, B**) *T. hamatum*; (**C, D**) and *T. velutinum* (**E, F**) on Potato dextrose Agar, Melin-Norkrans Medium (Müller et al., 2013) and Mandels Andreotti Medium (Mandels and Andreotti, 1978), respectively. Representative pictures are shown for each day. DAI indicates day after inoculation. The fungi grew in 21°C and permanent darkness except for the short period of scanning the petri dishes. The fungal diameter was measured daily, data are shown with mean ± SEM, n = 6, $P < 0.05$, One-Way ANOVA and LSD test.

(A)

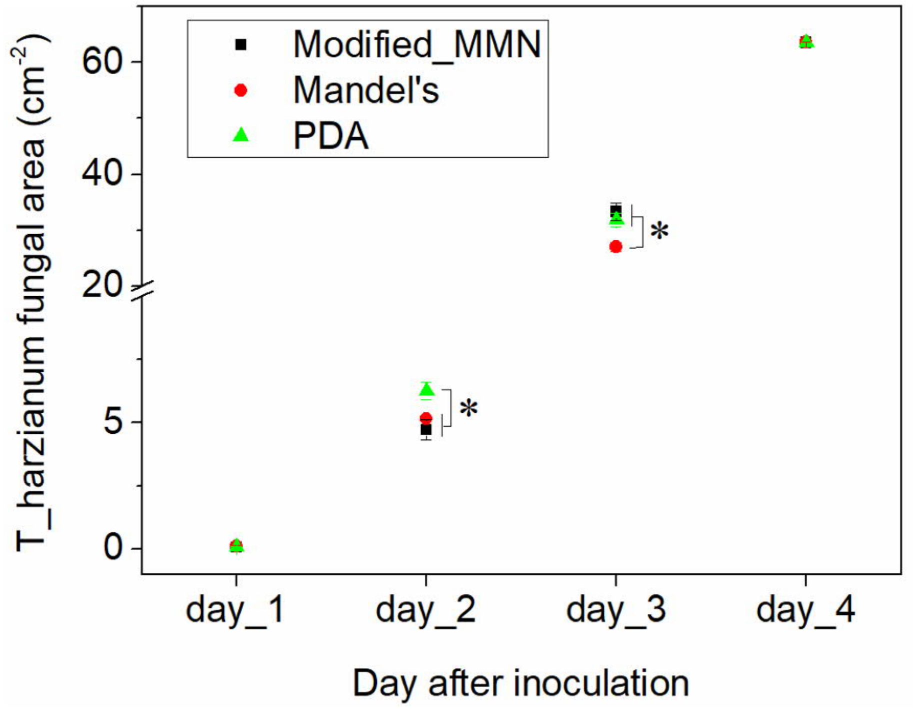

(B)

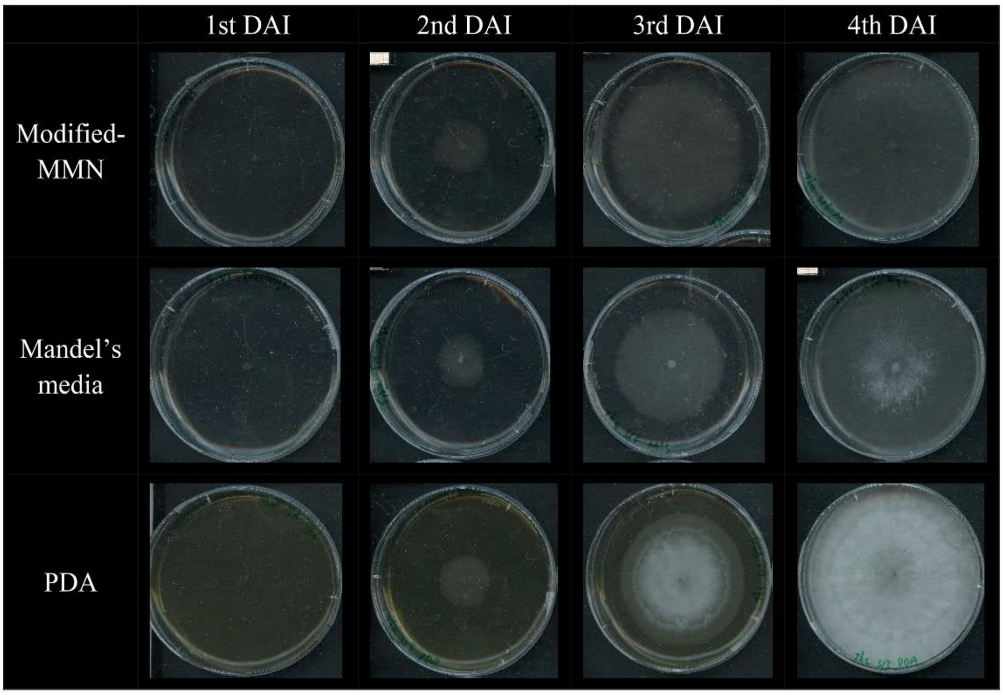

(C)

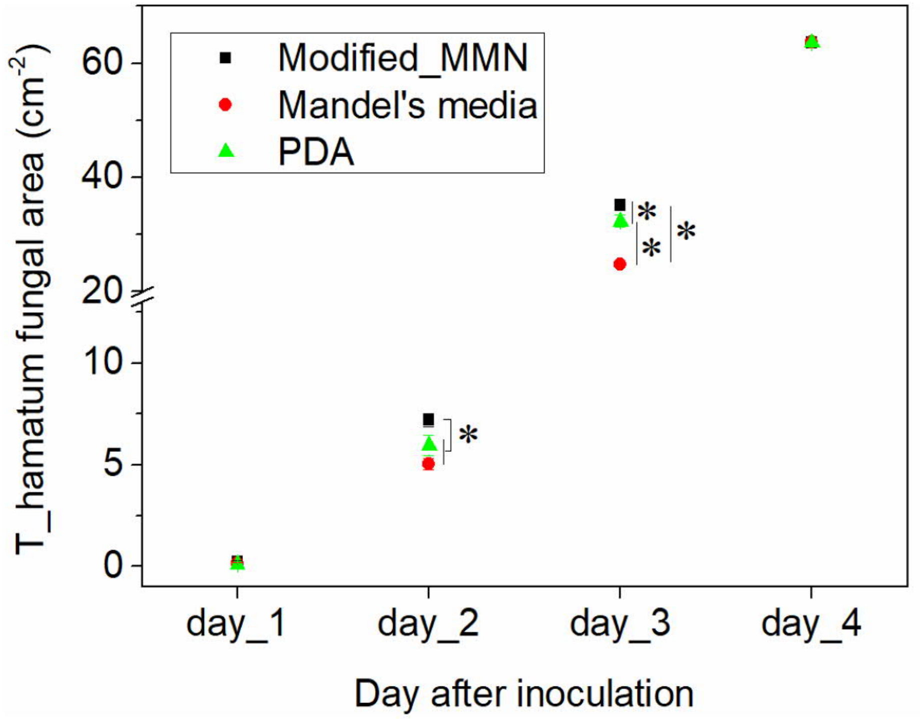

Modified_MMN
Mandel's media
PDA
T_hamatum fungal area (cm^{-2})
60
40
20
10
5
0
*
*
*
*
day_1
day_2
day_3
day_4
Day after inoculation

(D)

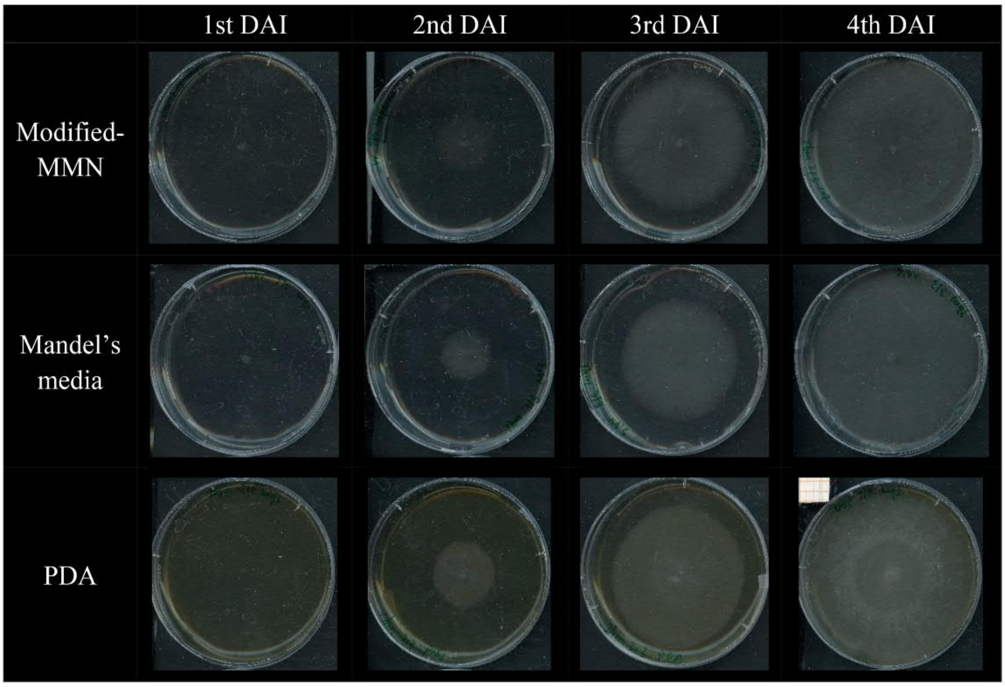

1st DAI
2nd DAI
3rd DAI
4th DAI
Modified-MMN
Mandel's media
PDA

(E)

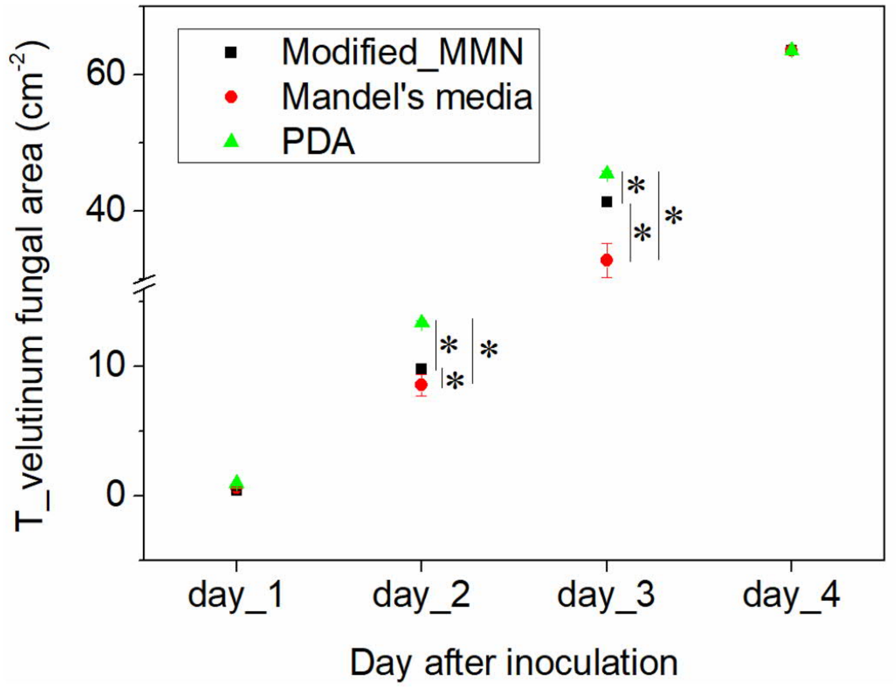

Modified_MMN
Mandel's media
PDA
T_velutinum fungal area (cm^{-2})
60
40
10
0
*
day_1
day_2
day_3
day_4
Day after inoculation

(F)

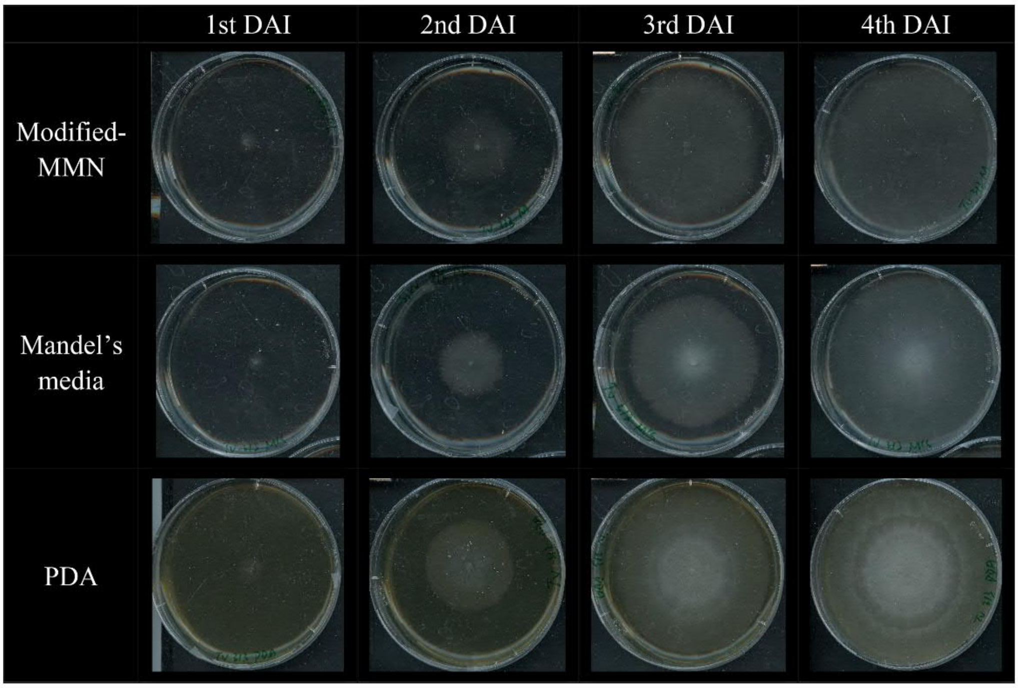

1st DAI
2nd DAI
3rd DAI
4th DAI
Modified-MMN
Mandel's media
PDA
