# Supplementary material for: Trichoderma Species Differ in Their Volatile Profiles and in Antagonism Toward Ectomycorrhiza Laccaria bicolor
Source: Front Microbiol. 2019 Apr 26;10:891. doi: 10.3389/fmicb.2019.00891 (PMC6499108; doi:10.3389/fmicb.2019.00891)
Supplement: Supplementary file 2 [file Data_Sheet_2.docx]

| **Supplementary Table S1.** Volatile organic compounds **(**VOCs; pmol cm^-2^ h^-1^) from *Trichoderma* species (*T. harzianum; Thz*, *T. hamatum; Thm* and *T. velutinum; Tv*) and *Laccaria bicolor* (*Lb*) in monoxenic cultures and from the different Trichoderma spp. co-cultured with *Laccaria*. MC, media contact in normal plate; AC, airborne contact in split-plate; DC, direct contact. Colors indicate VOC emission strengths; dark grey: very high (> 26 pmol cm^-2^ h^-1^), medium grey: high (6 to 13 pmol cm^-2^ h^-1^), grey: medium (1 to 5 pmol cm^-2^ h^-1^), light grey: low (0 to 1 pmol cm^-2^ h^-1^). SEM: standard error (n = 6); nd: not detected. The numbers refer to the compound names listed in Supplementary Table S2 | | | | | | | | | | | | | |
| --- | --- | --- | --- | --- | --- | --- | --- | --- | --- | --- | --- | --- | --- |
| **Compounds number** | **Lb** | **Thz** | **Thm** | **Tv** | **Thz-Lb MC** | **Thm-Lb MC** | **Tv-Lb MC** | **Thz-Lb AC** | **Thm-Lb AC** | **Tv-Lb AC** | **Thz-Lb DC** | **Thm-Lb DC** | **Tv-Lb DC** |
| ***Sesquiterpenes*** | |  |  |  |  |  |  |  |  |  |  |  |  |
| 1 | nd | nd | nd | nd | nd | nd | nd | nd | nd | nd | nd | 0.34±0.04 | nd |
| 2 | nd | nd | nd | nd | nd | nd | nd | nd | nd | nd | nd | 0.36±0.05 | nd |
| 3 | nd | nd | nd | nd | nd | nd | nd | nd | nd | nd | nd | 0.24±0.03 | nd |
| 4 | nd | nd | nd | nd | nd | nd | nd | nd | nd | nd | 1.97±0.50 | nd | nd |
| 5 | nd | nd | nd | nd | nd | nd | nd | nd | nd | nd | nd | 0.22±0.04 | nd |
| 6 | nd | nd | nd | nd | nd | nd | nd | nd | nd | nd | nd | 0.20±0.02 | nd |
| 7 | nd | nd | nd | nd | nd | nd | nd | nd | nd | nd | nd | 0.40±0.06 | nd |
| 8 | nd | 2.87±0.35 | nd | nd | 2.28±0.55 | nd | nd | 3.59±0.69 | nd | nd | 0.08±0.02 | nd | nd |
| 9 | nd | nd | 0.50±0.06 | 0.42±0.07 | nd | 0.28±0.06 | 0.29±0.10 | nd | nd | nd | nd | nd | nd |
| 10 | nd | nd | nd | nd | nd | nd | nd | nd | nd | nd | nd | 0.41±0.03 | nd |
| 11 | nd | 1.84±0.23 | nd | nd | 0.32±0.09 | nd | nd | 0.36±0.08 | nd | nd | 0.05±0.01 | nd | nd |
| 12 | nd | nd | nd | nd | nd | nd | nd | nd | nd | nd | nd | 2.43±0.30 | nd |
| 13 | nd | nd | 0.58±0.07 | 0.66±0.10 | nd | 0.57±0.06 | 0.48±0.11 | nd | nd | nd | nd | nd | nd |
| 14 | nd | nd | nd | nd | nd | nd | nd | nd | nd | nd | 0.34±0.08 | nd | nd |
| 15 | nd | 3.47±0.42 | nd | nd | 1.95±0.47 | nd | nd | 2.96±0.54 | nd | nd | nd | nd | nd |
| 16 | nd | 2.93±0.33 | nd | nd | 2.02±0.46 | nd | nd | 3.26±0.59 | nd | nd | 0.19±0.02 | nd | nd |
| 17 | nd | 6.50±0.97 | nd | 0.52±0.02 | 4.74±0.97 | nd | 0.43±0.04 | 7.79±1.24 | nd | 0.37±0.07 | 0.35±0.01 | 0.63±0.07 | nd |
| 18 | nd | nd | 0.78±0.10 | nd | nd | 0.99±0.16 | nd | nd | 0.75±0.06 | nd | nd | nd | nd |
| **Compounds number** | **Lb** | **Thz** | **Thm** | **Tv** | **Thz-Lb MC** | **Thm-Lb MC** | **Tv-Lb MC** | **Thz-Lb AC** | **Thm-Lb AC** | **Tv-Lb AC** | **Thz-Lb DC** | **Thm-Lb DC** | **Tv-Lb DC** |
| 19 | 0.39±0.03 | 0.21±0.03 | nd | nd | 0.47±0.12 | nd | 0.23±0.03 | 0.74±0.07 | nd | 0.29±0.03 | 0.06±0.01 | nd | 0.02±0.003 |
| 20 | nd | nd | nd | nd | nd | nd | nd | nd | nd | nd | nd | 1.44±0.15 | nd |
| 21 | nd | 2.10±0.26 | 2.61±1.26 | nd | 1.50±0.36 | 6.02±1.47 | nd | 2.45±0.47 | 3.19±0.50 | nd | 0.15±0.08 | nd | nd |
| 22 | nd | 1.38±0.12 | nd | nd | 0.70±0.15 | nd | nd | 1.10±0.26 | nd | nd | nd | nd | nd |
| 23 | nd | nd | nd | nd | nd | nd | nd | nd | nd | nd | nd | nd | 0.39±0.15 |
| 24 | nd | nd | 0.79±0.26 | nd | nd | 2.57±0.54 | nd | nd | 1.49±0.21 | nd | nd | 6.40±0.68 | nd |
| 25 | nd | 0.52±0.11 | nd | nd | 0.32±0.09 | nd | nd | 0.44±0.08 | nd | nd | nd | nd | nd |
| 26 | nd | nd | 0.26±0.10 | nd | 7.63±4.25 | 0.75±0.16 | nd | 9.37±2.75 | 0.41±0.05 | nd | 2.35±0.88 | 3.95±0.29 | 0.40±0.14 |
| 27 | nd | 0.79±0.21 | nd | nd | 0.85±0.16 | nd | nd | 1.33±0.23 | nd | nd | 2.49±0.52 | nd | nd |
| 28 | nd | nd | nd | nd | nd | nd | nd | nd | nd | nd | nd | 1.21±0.17 | nd |
| 29 | 1.37±0.15 | nd | nd | nd | 4.68±1.15 | nd | 0.62±0.09 | 7.92±1.38 | nd | 0.70±0.19 | 0.34±0.05 | nd | 0.04±0.01 |
| 30 | nd | 0.12±0.01 | nd | nd | 0.42±0.15 | nd | nd | 0.72±0.09 | nd | nd | 0.12±0.07 | nd | nd |
| 31 | nd | 0.28±0.03 | nd | nd | 0.22±0.06 | nd | nd | 0.28±0.06 | nd | nd | nd | nd | nd |
| 32 | nd | 0.30±0.06 | nd | nd | 0.15±0.04 | nd | nd | 0.17±0.02 | nd | nd | nd | 26.10±2.43 | nd |
| 33 | nd | nd | nd | nd | nd | nd | nd | nd | nd | nd | 0.15±0.02 | 0.15±0.01 | nd |
| 34 | nd | nd | nd | nd | nd | nd | nd | nd | nd | nd | nd | 0.11±0.01 | nd |
| 35 | nd | nd | nd | nd | nd | nd | nd | nd | nd | nd | nd | 0.15±0.02 | nd |
| 36 | nd | nd | nd | nd | nd | nd | nd | nd | nd | nd | 0.25±0.10 | nd | nd |
| ***Oxygenated Sesquiterpenes*** | | |  |  |  |  |  |  |  |  |  |  |  |
| 37 | nd | 0.36±0.06 | nd | nd | nd | nd | nd | nd | nd | nd | nd | nd | nd |
| 38 | nd | nd | nd | nd | nd | nd | nd | nd | nd | nd | 0.11±0.03 | nd | nd |
| ***Monoterpenes*** | |  |  |  |  |  |  |  |  |  |  |  |  |
| 39 | nd | nd | nd | nd | nd | nd | nd | nd | nd | nd | nd | 1.09±0.19 | nd |
| 40 | nd | nd | nd | nd | nd | nd | nd | 1.69±0.15 | nd | 1.41±0.62 | 0.37±0.06 | 0.25±0.05 | nd |
| **Compound number** | **Lb** | **Thz** | **Thm** | **Tv** | **Thz-Lb MC** | **Thm-Lb MC** | **Tv-Lb MC** | **Thz-Lb AC** | **Thm-Lb AC** | **Tv-Lb AC** | **Thz-Lb DC** | **Thm-Lb DC** | **Tv-Lb DC** |
| 41 | nd | nd | 12.66±1.79 | nd | nd | 11.79±2.06 | nd | nd | 9.70±0.64 | nd | nd | 0.15±0.02 | nd |
| ***oxygenated Monoterpene*** | | |  |  |  |  |  |  |  |  |  |  |  |
| 42 | nd | 42.87±6.16 | nd | nd | 44.56±8.26 | nd | nd | 53.45±11.6 | nd | nd | 10.21±4.23 | nd | nd |
| ***Other VOCs*** |  |  |  |  |  |  |  |  |  |  |  |  |  |
| 43 | nd | nd | nd | nd | nd | nd | nd | nd | nd | nd | 2.27±0.67 | nd | nd |
| 44 | nd | nd | nd | nd | 1.19±0.42 | nd | nd | 1.63±0.30 | nd | nd | 0.66±0.19 | nd | nd |
| 45 | nd | nd | nd | nd | nd | nd | nd | nd | nd | nd | 1.46±0.22 | nd | nd |
| 46 | 0.23±0.02 | nd | nd | nd | 0.23±0.13 | nd | nd | 0.16±0.03 | nd | nd | 0.56±0.14 | nd | nd |
| 47 | nd | nd | nd | nd | nd | nd | nd | nd | nd | nd | nd | 0.29±0.03 | nd |
| 48 | nd | nd | nd | nd | nd | nd | nd | nd | nd | nd | nd | 0.15±0.01 | nd |
| 49 | nd | 1.69±0.25 | nd | nd | 1.82±0.53 | nd | nd | 3.29±0.42 | nd | nd | nd | nd | nd |

**Supplementary Table S2.** Identification of all the VOCs detected during the experiment. The statistical parameters ‘identification quality’ (Qual), ‘confidence factor’ (K) and ‘cross-correlation coefficient’ (XCorr) are extracted from the probability-based matching (PBM) algorithm implemented in the software MSD Chemstation (Agilent Technologies, Palo Alto, CA, USA). RT, retention time; RI, non-isothermal Kovats retention index.

|  | | | | | | | |
| --- | --- | --- | --- | --- | --- | --- | --- |
| **Compounds** | **Compounds number** | **CAS registry number** | **RT (min)** | **RI** | **(Qual)** | **(K)** | **(XCorr)** |
| ***Sesquiterpenes*** |  |  |  |  |  |  |  |
| α-Cubebene | 1 | 17699-14-8 | 18.888 | 1368 | 99 | 133 | 9976 |
| Copaene | 2 | 3856-25-5 | 19.562 | 1402 | 97 | 120 | 9271 |
| β-Gurjunene | 3 | 17334-55-3 | 19.753 | 1411 | 96 | 114 | 9548 |
| β-Elemene | 4 | 515-13-9 | 19.772 | 1396 | 99 | 154 | 9184 |
| unknown SQT #3 | 5 |  | 19.81 | 1414 |  |  |  |
| Isoledene | 6 | 95910-36-4 | 19.886 | 1418 | 83 | 90 | 9891 |
| α-Gurjunene | 7 | 489-40-7 | 20.271 | 1437 | 99 | 155 | 9993 |
| α-Guaiene | 8 | 3691-12-1 | 20.398 | 1441 | 83 | 61 | 9759 |
| α-Cedrene | 9 | 469-61-4 | 20.464 | 1446 | 90 | 54 | 9394 |
| Germacrene D | 10 | 23986-74-5 | 20.484 | 1447 | 96 | 110 | 9817 |
| β-Curcumene | 11 | 28976-67-2 | 20.493 | 1480 | 91 | 84 | 9541 |
| Isocaryophyllene | 12 | 118-65-0 | 20.582 | 1452 | 98 | 136 | 9674 |
| Cedrene | 13 | 11028-42-5 | 20.617 | 1454 | 86 | 54 | 9340 |
| α-Bergamotene | 14 | 17699-05-7 | 20.626 | 1435 | 91 | 117 | 9959 |
| Thujopsene | 15 | 470-40-6 | 20.648 | 1445 | 86 | 96 | 9111 |
| Valencen | 16 | 4630-07-3 | 20.67 | 1466 | 83 | 100 | 9742 |
| γ-Muurolene | 17 | 30021-74-0 | 20.741 | 1459 | 81 | 80 | 9053 |
| Zingiberene | 18 | 495-60-3 | 20.781 | 1461 | 89 | 83 | 9646 |
| **Compounds** | **Compounds number** | **CAS registry number** | **RT (min)** | **RI** | **(Qual)** | **(K)** | **(XCorr)** |
| Acoradiene | 19 | 24048-44-0 | 21.074 | 1474 | 80 | 90 | 9690 |
| Eremophilene | 20 | 10219-75-7 | 21.625 | 1500 | 95 | 105 | 9265 |
| γ-Candinene | 21 | 39029-41-9 | 21.658 | 1502 | 86 | 102 | 9327 |
| β-Himachalene | 22 | 1461-03-6 | 21.769 | 1510 | 95 | 108 | 8869 |
| (+)-Ledene | 23 | 21747-46-6 | 22.113 | 1521 | 86 | 103 | 9110 |
| β-Selinene | 24 | 17066-67-0 | 22.12 | 1522 | 99 | 135 | 9960 |
| β-Bisabolene | 25 | 495-61-4 | 22.213 | 1507 | 96 | 96 | 9647 |
| α-Selinene | 26 | 473-13-2 | 22.248 | 1527 | 96 | 116 | 9340 |
| γ-Selinene | 27 | 515-17-3 | 22.279 | 1522 | 96 | 109 | 9398 |
| β-Cadinene | 28 | 523-47-7 | 22.341 | 1531 | 80 | 82 | 9645 |
| Cadine-1,4-diene | 29 | 16728-99-7 | 22.488 | 1533 | 91 | 94 | 9562 |
| unknown SQT #1 | 30 |  | 22.528 | 1539 |  |  |  |
| β-Sesquiphellandrene | 31 | 20307-83-9 | 22.607 | 1534 | 91 | 101 | 7142 |
| δ-Cadinene | 32 | 483-76-1 | 22.771 | 1526 | 95 | 114 | 9159 |
| Selina-3,7(11)-diene | 33 | 6813-21-4 | 22.86 | 1527 | 94 | 86 | 9944 |
| Cubenene | 34 | 29837-12-5 | 22.966 | 1558 | 98 | 119 | 9959 |
| α-Cadinene | 35 | 24406-05-1 | 23.055 | 1561 | 98 | 133 | 9640 |
| unknown SQT #2 | 36 |  | 26.503 | 1691 |  |  |  |
| ***oxygenated Sesquiterpenes*** | |  |  |  |  |  |  |
| Trichoacorenol | 37 | 61050-89-3 | 26.649 | 1673 | 99 | 166 | 9527 |
| 1,4-trans-1,7-cis-Acorenone | 38 | 39510-36-6 | 27.332 | 1632 | 95 | 117 | 8999 |
| ***Monoterpenes*** |  |  |  |  |  |  |  |
| β-Myrcene | 39 | 127-91-3 | 11.365 | 997 | 96 | 98 | 9986 |
| Limonene | 40 | 138-86-3 | 12.367 | 1032 | 98 | 122 | 9970 |
| **Compounds** | **Compounds number** | **CAS registry number** | **RT (min)** | **RI** | **(Qual)** | **(K)** | **(XCorr)** |
| γ-Terpinene | 41 | 99-85-4 | 12.446 | 1049 | 91 | 66 | 9860 |
| ***oxygenated Monoterpene*** | |  |  |  |  |  |  |
| Tetrahydrocarvone | 42 | 499-70-7 | 14.52 | 1207 | 50 | 40 | 9856 |
| ***Other VOCs*** |  |  |  |  |  |  |  |
| 3-Octanone | 43 | 106-68-3 | 11.265 | 989 | 95 | 83 | 9932 |
| Cyclohexane, 1,2,4-tris(methylene)- | 44 | 14296-81-2 | 12.607 | 1056 | 78 | 89 | 8997 |
| 1,3-Octadiene | 45 | 1002-33-1 | 8.9 | 826 | 87 | 73 | 9541 |
| (+)-Cuparene | 46 | 16982-00-6 | 22.576 | 1517 | 93 | 89 | 9823 |
| (-)-Calamenene | 47 | 483-77-2 | 22.7 | 1547 | 98 | 108 | 9990 |
| α-Calacorene | 48 | 21391-99-1 | 23.223 | 1568 | 91 | 106 | 9682 |
| unknown #1 | 49 | 286-62-4 | 23.64 | 1585 |  |  |  |
